# The Validity and Reliability of Live Football Match Statistics From Champdas Master Match Analysis System

**DOI:** 10.3389/fpsyg.2019.01339

**Published:** 2019-06-11

**Authors:** Bingnan Gong, Yixiong Cui, Yang Gai, Qing Yi, Miguel-Ángel Gómez

**Affiliations:** ^1^China Football College, Beijing Sport University, Beijing, China; ^2^Facultad de Ciencias de la Actividad Física y del Deporte, Universidad Politécnica de Madrid, Madrid, Spain; ^3^AI Sports Engineering Lab, School of Sports Engineering, Beijing Sport University, Beijing, China

**Keywords:** performance analysis, reliability, validity, football, match statistics

## Abstract

The aim of the present study was to investigate the validity of match variables and the reliability of *Champdas Master System* used by trained operators in live association football match. Twenty professional football coaches voluntarily participated in the validation of match variables used in the System. Four well-trained operators divided into two groups that independently analyzed a match of Spanish La Liga. The Aiken’s V averaged at 0.84 ± 0.03 and 0.85 ± 0.03 for the validation of indicators. The high Kappa values (Operator 1: 0.92, 0.90; Operator 2: 0.91, 0.88), high intra-class correlation coefficients (varied from 0.93 to 1.00), and low typical errors (varied from 0.01 to 0.34) between the first and second data collection represented a high level of intra-operator reliability. The Kappa values for the inter-operator reliability of were 0.97 and 0.89. The intra-class correlation coefficients and typical errors ranged from 0.90 to 1.00 and ranged from 0.01 to 0.24 for two independent operators within two data collections. The results suggest that the *Champdas Master system* can be used validly and reliably to gather live football match statistics by well-trained operators. Therefore, the data obtained by the company can be used by coaches, managers, researchers and performance analysts as valid match statistics from players and teams during their professional tasks and investigations.

## Introduction

Sport performance analysis during actual competition is one of the main sources of information that are beneficial for the training and coaching process (Sampaio and Leite, 2013). In essence, coaches and athletes could be provided with information of interest that is difficult to be detected throughout their subjective perception, and then refine their training planning and improve athlete’s performance purposefully (Hughes and Franks, 2015). Within its domain, the analysis of technical and tactical performance has captured substantial research interest in either individual sports (Reid et al., 2016; Cui et al., 2019), or team sports (Zhang et al., 2018; Zhou et al., 2018), where a set of performance indicators that contains relevant information about players and teams’ match performance during competition have been established and collected via observational approaches (Hughes and Bartlett, 2002; Lames and McGarry, 2007; Hughes and Franks, 2015). Fundamentally, it is mainly based on systematic observation, understood as an organized recording and quantification of sport behaviors in their natural context (O’Donoghue and Mayes, 2013; Sampaio and Leite, 2013). To achieve this purpose, both a well-designed notational system and valid, precise and objective performance indicators are required so that technical-tactical aspects of match performance could be easily gathered and used for the subsequent analysis and practical applications (Bradley et al., 2007; Carling et al., 2007; O’Donoghue, 2007).

Therefore, as a prerequisite for any performance analysis research that uses novel system or instrument, the repeatability and accuracy of this new tool, and the validity of performance indicators used should be validated, before collecting and analyzing players and teams’ performances (O’Donoghue, 2014; Chacón-Moscoso et al., 2018). Despite that currently there are some automatic player tracking system available for performance analysis, most of observational studies or practices in sport field are still done with computerized notational systems where performance analysts are required to manually code sport performance indicators with predetermined short-cut keys (Bradley et al., 2007; Hizan et al., 2010; Liu et al., 2013; Beato et al., 2018). From a theoretical and applied perspective, a performance indicator should help to explain the match outcome; and thus advance understanding, providing for meaningful insights of game behavior (O’Donoghue, 2009). The use of precise operational definitions and the validity of performance indicators are related to reliability of data collection in performance analysis and therefore have a strong impact on the correct interpretation of match performances (McGarry, 2009).

Validity is generally referred to as the ability of a measurement tool to reflect what it is designed to measure (Atkinson and Nevill, 1998), and usually for performance analysis instrument, it can be determined through expert coaches’ opinions in each sports category (Hraste et al., 2008; Cupples and O’Connor, 2011; Larkin et al., 2016; Torres-Luque et al., 2018). For instance, Larkin et al. (2016) validated a coding instrument of assessing movement awareness and technical skills for soccer players with a panel of nine experts. Similarly, with the review and confirmation of eleven experts, Torres-Luque et al. (2018) validated an observational instrument for analyzing the technical-tactical match performance in tennis. Further, the reliability of a sport notational system is as important as its validity (Hayen et al., 2007). It refers to the reproducibility of values of a test, assay or other measurement in repeated trials on the same individuals (intra-observer reliability) (O’Donoghue, 2009), and repeatability over different observers (inter-observer reliability) (Hopkins, 2000). Sports notational system may be limited in reliability due to manual errors, observer’s inexperience, number of observers (Beato et al., 2018) so that its results will mislead coaches or performance analysts to make poor decisions about training and match preparation.

In recent years, the development of semi-automatic match analysis systems in elite football has enhanced the accessibility to match information related to match events and movements (Carling et al., 2007; Mackenzie and Cushion, 2013). And as a result, performance directors, coaches and researchers frequently utilize these systems to gain insights into football match performance. Concurrently, the accuracy and reliability of some widely used systems from various commercial football match statistics providers have been validated and verified, such as AMISCO^®^ system (Carling et al., 2008; Zubillaga et al., 2009; Lago et al., 2010; Dellal et al., 2011; Castellano et al., 2014), PROZONE Sports Ltd.^®^ (Bradley et al., 2007, 2011; Castellano et al., 2014), SportsCode (Hughes, 2004; Reed and Hughes, 2006; O’Donoghue and Holmes, 2014), OPTA Sportsdata (Oberstone, 2009, 2010, 2011; Liu et al., 2013), SICS (Rampinini et al., 2009; Osgnach et al., 2010; Beato and Jamil, 2017), Dartfish (Eltoukhy et al., 2012; Padulo et al., 2015; Larkin et al., 2016; Li et al., 2016), and Nacsports (Clear et al., 2017). Indeed, these systems have presented both coder-friendly operating platform and high reliability in measuring technical-tactical performance indicators. Yet, there remain some limitations concerning the above-mentioned studies and measures. Most of them only studies the test-retest reliability of these systems, not considering the content validity of performance events or indicators included. Besides, some systems were mainly focused on the successful or unsuccessful outcome of technical performance events, such as shoots, dribbles, crosses (Larkin et al., 2016), so that tactical information related to pass directions and network is not able to be gathered, which helps to understand complex match characteristics of this invasion sport and becomes one of the recent research topics for football investigators (Gonçalves et al., 2017; Pollard, 2019; Praça et al., 2019).

In comparison with the previous existing systems, *Champdas Master System*, a semi-automatic match analysis system developed by the Champion Technology Co., Ltd., (a leading Chinese sport data company founded in 2004), has been employed to provide match data services for the majority of professional teams from Chinese Football Super League (first division) and Chinese Football Association China League (second division), China National Youth Super League (U13–U19 divisions), China Men’s National Team, clubs from Korean K-league (first division). Meanwhile, the company has also been cooperating with major Chinese online sport video media (PPTV), by collecting, storing, analyzing and visualizing professional football match data of the first leagues in Korea, Spain and United Kingdom (first leagues) during online match broadcasting. In a word, most of match reports and analyses provided by the system are widely used by Asian professional football clubs, coaches, media, and governing organizations. Furthermore, what stands the system out is that it not only allows for common match performance indicators as its peers do, but also includes a more complex classification of players’ passing directions, which is seldom found in other systems. As noted above, passing behaviors reveal great extent of tactical information about football, because some teams try to create opportunities by long direct passes, whereas other teams are characterized by possession-style type of play (Larkin et al., 2016; Goes et al., 2019). Players are most likely more successful when passing the ball backward or sideways than attempting forward passes, but the latter has been regarded as a key performance indicator when evaluating penetration of offensive actions and assessing players’ performance (Goes et al., 2019). However, given a wide range of professional leagues, clients and audience it serves, little is known about whether match performance indicators used in the *Champdas Master System* are valid and the live match data collecting process is reliable among its trained operators.

Consequently, it is imperative to execute a thorough validity and reliability analysis of the system, so that its statistics would be trustworthy for research, coaching and broadcasting purposes. Therefore, the present study was aimed: (i) to identify the validity of match performance variables used by *Champdas Master System*; (ii) to verify the intra- and inter-operator reliability of *Champdas Master System* used by well-trained operators to collect match statistics in live association football match.

## Materials and Methods

### Validity of Performance Variables Used by *Champdas Master System*

At the first stage of the study, a panel of experts constituted by 20 coaches or assistant coaches from China, Spain, Portugal, Germany, and Ireland voluntarily participated and completed the questionnaire that was aimed to validate the performance variables used in *Champdas Master System*. The inclusion criteria of the coaches were as follows: (i) Having coached professional teams of a level equivalent to the first and second divisions in Asian Football Confederation (AFC) or Union of European Football Associations (UEFA) or coached in a semi-professional level, equivalent to AFC or UEFA third division; and (ii) Owning coach licenses equivalent or higher than AFC-B or UEFA-A level. The participating coaches had an average coaching experience of 13.3 ± 7.1 years, and among twenty of them, five had UEFA-Pro license, five had UEFA-A license, nine had AFC-A license and one had AFC-B license. Prior to the filling of questionnaire and sign the voluntary informed consent, the study purpose and the anonymously academic use of their answers were explained to each coach.

### Performance Variables and Operational Definitions

**FIGURE 1:**
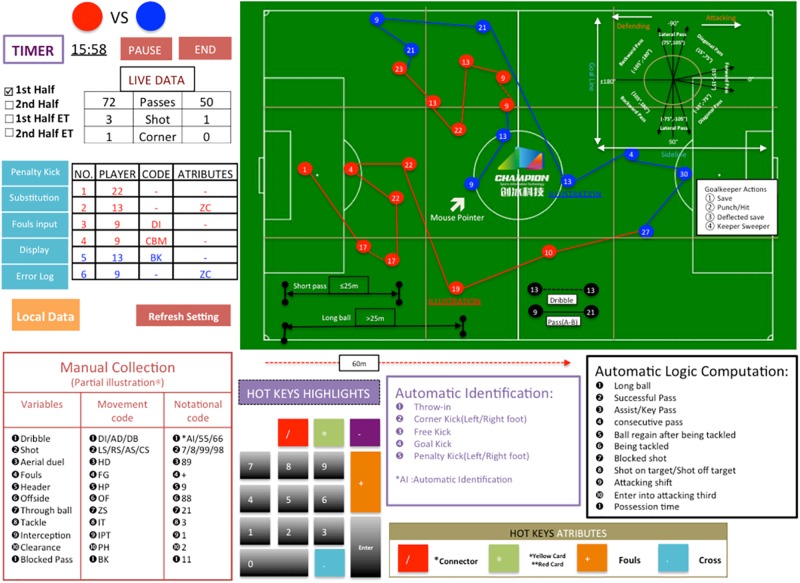
Illustration of *Champdas Master System* operation interface diagram.

The questionnaire was based on performance variables used by *Champdas Master System* and they were divided into three domains: (i) Attacking-related performance; (ii) Passing-related performance, and (iii) Defending-and-Goalkeeper-related performance. The criterion of selecting these variables were mainly based on two categories of existing literature, namely, studies that examined the validity of other match analysis systems and analyzed variables (Valter et al., 2006; Bradley et al., 2007; Liu et al., 2013; Castellano et al., 2014; Beato et al., 2018); and studies that focused on tactical patterns and passing behaviors of football players (Rein et al., 2017; Goes et al., 2019; Pollard, 2019). Within all variables, the inclusion of two variables should be highlighted. First of all, considering the width of the pitch and different pitch paths (left, center, and right) and their actual usefulness in interpreting offensive behaviors (Sgrò et al., 2017), we included the “attacking shift” as a performance variable, revealing a quick ball transition from side to side. This corroborates with the findings of Rein et al. (2017) in that more successful teams could increase space control in the attacking zone through passing, creating defensive disadvantages for the opposing teams. Secondly, passing directions were established calculating the angles from current passes to the next events in relation to the sideline and attacking direction (see Figure 1; Serpiello et al., 2017; Goes et al., 2019).

#### Attacking-Related Performance

##### Attacking shift

The situation takes place in midfield or attacking third where the attacking players take the initiative to transfer the ball from one sideway to the other (done with no more than three passes), for creating better attacking space.

##### Enter into attacking third

(Pitch is divided into three zones, including attacking third, middle third and defending third. Enter into offensive zone means entering into attacking third). It includes the following conditions: (1) After players enter into the attacking third, a transition of ball possession is realized (including possession gained by defensive actions or opponent’s turnover); (2) A dead ball occurs.

##### Dribble

A dribble is an attempt by a player to beat an opponent in possession of the ball. A successful dribble means the player beats the defender while retaining possession; unsuccessful ones are where the dribbler is tackled.

##### Shot

An attempt to score a goal, made with any (legal) part of the body, either on or off target. The outcomes of shot could be: goal, shot on target, shot off target, blocked shot, post.

##### Shot on target

The definition of a “shot on target” or a “shot on goal” (SOG) is goal or any shot attempt to goal, which required intervention to stop it going in or resulted in a goal if left unblocked.

##### Possession gained

The total number of possession regained by active defending (tackle, interception), or by passive recovery (ball cleared by the opponents).

##### Aerial Duel

Aerial Duel can be also called as heading duel. Two players competing for a ball in the air, for it to be an aerial both players must jump and challenge each other in the air and have both feet off the ground. The player who wins the duel gets the Aerial Won, and the player who doesn’t get an Aerial Lost.

#### Passing-Related Performance

##### Pass

Any pass attempted from one player to another. Excluding free kicks, corners, throw-ins, and goal kicks.

##### Successful pass

Any pass successfully reached from one player to another. Excluding free kicks, corners, throw-ins, and goal kicks.

##### Forward pass

Forward pass (The angle between pass direction and the parallel of sideline is <15°).

##### Through ball

Ball passed through the last line of defense.

##### Lateral pass

The lateral pass to the left or right (the angle between pass direction and the parallel of goal line ≤15°).

##### Diagonal pass

The pass with the angle between pass direction and the parallel of sideline is {(15°, 75°), (105°, 165°)}.

##### Backward pass

The pass with the angle between backward pass route and side line ≤75°.

##### Long pass

The distance of pass >25 m.

##### Short pass

The distance of pass ≤25 m.

##### Assist

The final pass or cross leading to goal-scoring.

##### Consecutive pass

The total number of passes that a team realizes without losing ball possession or causing a dead ball.

##### Key pass

The final pass or cross leading to the recipient of the ball that attempts to make a goal, but fails.

##### Cross

Any pass that delivers the ball into the penalty area by the attacking team, from lateral areas of the attacking third (not played inside of the penalty area).

#### Defending-and-Goalkeeper-Related Performance

##### Tackle

When the opposing player is in possession of the ball, but has no intention to pass, the defending player acts dispossessing the ball. A tackle won is given during following conditions: when a player makes a tackle and possession is retained by either himself or one of his teammates; when the tackle results in the ball leaving the field of play.

##### Interception

When a player intercepts any pass event between opposition players and prevents the ball reaching its target. Note: Defending player should be close to the receiver.

##### Clearance

Player under pressure gets the ball clear of the danger zone or/and out of play. If the ball is intentionally played into another teammate, it is not considered as a clearance, but a pass.

##### Blocked pass

Similar to interception but the opposing player is already very close to ball and successfully block the pass from passing player. It usually happens when player consciously or unconsciously blocks the pass immediately when the ball is attempted from one player to another.

##### Blocked shot

A defensive block, blocking a shot going on target. This must be awarded to the player who blocks the shot.

##### Save

The goalkeeper prevents the ball from entering the goal with any part of his body.

##### Punch

The goalkeeper punches/hits any ball played into the box.

##### Deflected save

When goalkeeper saves a shot, but does not catch the ball.

##### Keeper sweeper

When the goalkeeper runs out from the goal line to either intercept a pass or close down an attacking player.

### Questionnaire Design and Quantitative Evaluation

In order to make sure that variables were precisely defined and validly represented certain aspect match behavior (O’Donoghue, 2007), the questionnaire was designed to quantitatively evaluate: (i) the level of correct definitions of the performance variables, and (ii) the level of variable pertinence to match behaviors. This evaluation was comprised of a scale from 1 to 10, and an example of the questionnaire is presented in Table 1. There was no time limit to complete the questionnaire and the average time the coaches used to fill out the questionnaire was 20 min.

**Table 1:** Illustration of questionnaire sent to experts.

**Dribble**
(1) Definition: A dribble is an attempt by a player to beat an opponent in possession of the ball. A successful dribble means the player beats the defender while retaining possession; unsuccessful ones are where the dribbler is tackled
**Poorly defined** 1-2-3-4-5-6-7-8-9-10 **Correctly defined**
(2) Pertinence: Does this variable seem pertinent to the match performance?
**None** 1-2-3-4-5-6-7-8-9-10 **Maximum**

Afterward, their answers were collected and analyzed to calculate the content validity for each variable. To achieve this, the Aiken’s *V* coefficient of each item and its respective 95% confidence interval were used (Aiken, 1980; Penfield et al., 2004). The magnitude of this coefficient was from 0 to 1, with 1 being the greatest possible magnitude that indicates a perfect agreement among the judges regarding the highest validity score “10” of the contents evaluated in the scale. An item was determined to be valid if its Aiken’s *V* coefficient exceeds the exact critical value calculated by the following formula that takes into account the number of judges and items in the questionnaire sample was used (Aiken, 1985):

$$\bar{V}=0.5+\left(z.2\sqrt{\frac{3mn(c-1}{c+1}}\right)$$

where *z* is the level of significance, *m* the number of items that the experts should evaluate, *n* is the number of expert judges that participate in the study, and *c* the maximum value that can evaluate an item. The exact critical value was then calculated to be 0.52 via the formula at a statistical significance level of *p* < 0.05.

### Intra- and Inter-Operator Reliability Test for the *Champdas Master System*

*Champdas Master System* is a computerized match analysis system developed by Champion Technology Co., Ltd., to generate live match statistics for professional association football matches. Any performance analyst using the System has to firstly go through a rigorous learning process to get comprehensively familiarized with the definitions of all match actions or events, live coding mode, hot-keys, on-screen manual positioning with mouse, and movement characteristics (see the illustration in Figure 1). Later, they are required to practice the learned knowledge and skills within various trial matches so as to be capable of collecting formal live match statistics.

Main data capture mode combines hot-keys of keyboard and on-screen positioning to represent events and labels. The on-screen positioning functions by using mouse markings on a scaled-down version of football pitch, which is employed for tracking players. The movements of mouse and codes correspond to the actual actions performed by players in the actual match.

Event buttons/labels represent match events that are to be recorded over the course the match. Some events may have multiple levels of information, so that some short-cut keys or combination of keys recording different aspects of the same event are used and synchronized by the system.

Two categories of data source are automatically input into the system once manually coding match events. The first category includes corner, free kick and throw-in, etc., events that can be automatically identified given the marking locations on the simulated pitch; the other category is composed of long ball, successful pass, consecutive pass, attacking shift, etc., actions that can be automatically and logically generated from the relationship of players and pitch zones. The corresponding time of the event is also recorded automatically once an event is notated. Meanwhile, the marked event locations would be later integrated to generate additional tactical performance information.

### Live Data Collection and Sampled Match

To test the system reliability, four well-trained operators (experience = 1.5, 1.5, 2, and 2 years) from Champion Technology Co., Ltd., collected twice (with an interval of 2 weeks) the match data of the 19th round of Spanish La Liga Santander between Real Madrid and Villarreal contested in January 13, 2018 (Live broadcast from a conventional TV coverage). The number of match used and the coding procedure followed the routine of previous studies that validated similar systems (Bradley et al., 2007; Choi et al., 2007; Liu et al., 2013; Beato et al., 2018).

The operators were separated into two groups and observed the match independently. To be capable of formally operating the system, new operators should take part in a training process that consists of five parts: (i) definition learning, (ii) actions coding, (iii) practice in test server, (iv) played-match coding, and (v) live match coding (Champion Technology Co., Ltd., 2018). Normally, during months of training process, new operators were required to get familiar with all match actions, events and corresponding codes, and gradually develop the accuracy and proficiency in data collection. During the live match coding, there was only one principal operator from each group who was in charge of coding all match events. While the other was responsible for checking the completeness of whole dataset, amending any major inconsistence of statistics wherever needed, and then the final report was usually regarded as one-operator work. Therefore, in the current study, Operator 1 and Operator 2 were used to represent each coding group. A total of 27 players were observed, which included 22 starters, 5 substitutes, and 2 goalkeepers. The data collection was officially authorized and supported by Champion Technology Co., Ltd., and the institutional ethics committee from the Technical University of Madrid approved the study.

After the data collection, the raw data were output into Microsoft Excel with their corresponding timeline. As there were large differences in players’ action counts due to different on-field time, the agreement of match actions and events coded by independent operators was analyzed by considering the same three number of groups used during validation stage: (i) attacking related actions: dribble won, dribble lost, corner, attacking shift, possession gained, free kick, goal, header, shot, shot on target, shot off target, shot saved, throw-in, offside, and enter into attacking third; (ii) passing-related actions: pass, successful pass, forward pass, through ball, lateral pass, diagonal pass, backward pass, long pass, short pass, assist, consecutive pass, key pass, and cross; and (iii) defending and goalkeeper related actions: blocked pass, blocked shot, clearance, interception, tackle won, tackle lost, aerial won, aerial lost, yellow card, keeper sweeper, save, punch, and deflected save.

### Statistical Analysis

The intra- and inter-operator reliability of collected match statistics were determined using weighted *Kappa* statistic (Altman, 1990; O’Donoghue, 2010), mean, change in the mean, standardized typical error (TE) and intra-class correlation (ICC) (Hopkins, 2000). The *kappa* statistic was interpreted according to Altman’s evaluation scheme (Altman, 1990): κ ≤ 0.2 poor agreement; 0.2 ≤ κ < 0.4 fair agreement; 0.4 ≤ κ < 0.6 moderate agreement; 0.6 ≤ κ < 0.8 good agreement; κ ≥ 0.8 very good agreement. The value of standardized typical error should be doubled and the thresholds for the levels of disagreement are as follow: < 0.20 trivial; 0.21–0.60 small; 0.61–1.20 moderate; 1.21–2.00 large; 2.01–4.00 very large; >4.00 extremely large (Hopkins, 2000; Smith and Hopkins, 2011). The mean, change in the mean and standardized TE and ICC were calculated using the spreadsheet developed by Hopkins (2000).

## Results

Based on the evaluation of twenty professional coaches over 31 variables, the result of Aiken’s V averaged at 0.84 ± 0.03 for the degree of variable pertinence to match performance and 0.85 ± 0.03 for the correct definition of variable, showing high values in relation to content validity of all variables (see Table 2).

**Table 2:** Evaluation by 20 expert judges of the pertinence and definition of performance variables.

	Pertinence to match performance			Correct definition of variable		
	Mean (SD)	Aiken’s V	95% CL	Mean (SD)	Aiken’s V	95% CL
Attacking shift	8.3 (1.4)	0.81	0.806–0.815	8.2 (1.6)	0.79	0.789–0.799
Enter into attacking third	8.5 (1.6)	0.83	0.823–0.832	8.4 (1.7)	0.82	0.817–0.826
Possession time	8.9 (1.8)	0.87	0.868–0.875	9.1 (1.6)	0.89	0.89–0.897
Dribble	8.5 (1.8)	0.83	0.823–0.831	8.7 (1.5)	0.85	0.845–0.854
Shot	9.3 (1.2)	0.92	0.918–0.925	9.4 (1.0)	0.93	0.929–0.936
Shot on target	9.0 (1.4)	0.88	0.878–0.887	9.0 (1.3)	0.88	0.878–0.887
Possession gained	9.0 (1.3)	0.89	0.884–0.892	9.1 (1.1)	0.90	0.895–0.903
Aerial Duel	8.5 (2.0)	0.83	0.823–0.831	8.6 (1.7)	0.84	0.84–0.848
Pass	8.5 (2.8)	0.83	0.824–0.831	8.8 (2.4)	0.87	0.863–0.869
Successful pass	8.6 (2.5)	0.84	0.841–0.847	8.7 (2.4)	0.86	0.852–0.858
Forward pass	9.0 (1.6)	0.88	0.879–0.887	9.1 (1.4)	0.89	0.89–0.898
Through ball	8.4 (2.4)	0.82	0.818–0.825	8.6 (2.3)	0.84	0.835–0.842
Lateral pass	8.7 (1.7)	0.85	0.845–0.854	8.7 (1.6)	0.86	0.851–0.859
Diagonal pass	8.7 (1.7)	0.85	0.845–0.854	8.7 (1.5)	0.86	0.851–0.859
Backward pass	8.5 (1.8)	0.83	0.823–0.831	8.7 (1.6)	0.85	0.845–0.854
Long pass	8.7 (1.8)	0.85	0.845–0.853	8.8 (1.7)	0.86	0.856–0.865
Short pass	8.8 (1.9)	0.87	0.862–0.87	9.0 (1.6)	0.88	0.879–0.886
Assist	8.9 (2.5)	0.87	0.869–0.875	8.9 (2.4)	0.88	0.874–0.88
Consecutive pass	8.5 (2.5)	0.83	0.824–0.831	8.5 (2.4)	0.83	0.829–0.836
Key pass	8.5 (2.5)	0.83	0.83–0.836	8.6 (2.4)	0.84	0.841–0.847
Cross	8.4 (1.5)	0.82	0.817–0.826	8.5 (1.5)	0.83	0.828–0.837
Tackle	8.5 (1.5)	0.83	0.823–0.832	8.5 (1.4)	0.83	0.828–0.837
Interception	8.9 (1.1)	0.87	0.867–0.876	9.0 (0.9)	0.89	0.883–0.893
Clearance	8.8 (1.5)	0.87	0.862–0.87	8.9 (1.3)	0.88	0.873–0.881
Ball regain after being tackled	8.3 (2.5)	0.81	0.807–0.814	8.5 (2.3)	0.83	0.829–0.836
Blocked pass	8.3 (2.4)	0.81	0.807–0.814	8.5 (2.2)	0.83	0.824–0.831
Blocked shot	8.7 (2.3)	0.85	0.846–0.853	8.7 (2.4)	0.85	0.846–0.853
Save	8.6 (2.4)	0.84	0.835–0.842	8.6 (2.3)	0.84	0.84–0.847
Punch	8.3 (2.7)	0.81	0.802–0.808	8.5 (2.4)	0.83	0.829–0.836
Deflected save	8.4 (2.3)	0.82	0.818–0.825	8.6 (2.3)	0.84	0.835–0.842
Keeper sweeper	8.6 (2.4)	0.84	0.835–0.842	8.6 (2.3)	0.84	0.84–0.847

**FIGURE 2:**
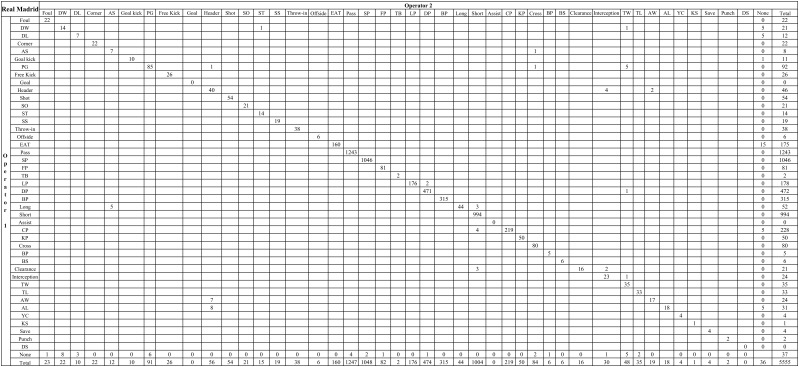
Frequency of Real Madrid’s events recorded by the two independent operators in two collections. DW, dribble won; DL, dribble lost; AS, attacking shift; PG, possession gained; SO, shots off target; ST, shot on target; SS, shot saved; EAT, enter into attacking third; SP, successful pass; FP, forward pass; TB, through ball; LP, lateral pass; DP, diagonal pass; BP, backward pass; CP, consecutive pass; KP, key pass; BP, blocked pass; BS, blocked shot; TW, tackle won; TL, tackle lost; AW, aerial won; AL, aerial lost; YC, yellow card; KS, keeper sweeper; DS, deflected save.

Table 3 showed that there were in total 5,430 events agreed by two independent operators within two collections for Real Madrid, and 4,065 for Villarreal. Comparing intra-operator data collections between the first and second collection, the average time difference of event-coding was 0.91 ± 0.94 s for Operator 1 and 0.81 ± 0.88 s for Operator 2, respectively. While average time difference was 0.89 ± 0.88 s between Operator 1 and Operator 2 for inter-operator data collections. Details can be seen from Figure 2, 3. The *Kappa* statistics for the events of two teams were 0.97 and 0.89, showing a very good agreement between independent operators.

**Table 3:** Number of events coded by different operators during two data collections.

	Agreement	Operator 1		Operator 2		*Kappa*
		Total	Disagreed	Total	Disagreed	
Real Madrid	5,430	5,518	88	5,519	89	0.97
Villarreal	4,065	4,393	328	4,391	326	0.89

*All event numbers presented in the table are sum of two data collections.*

**FIGURE 3:**
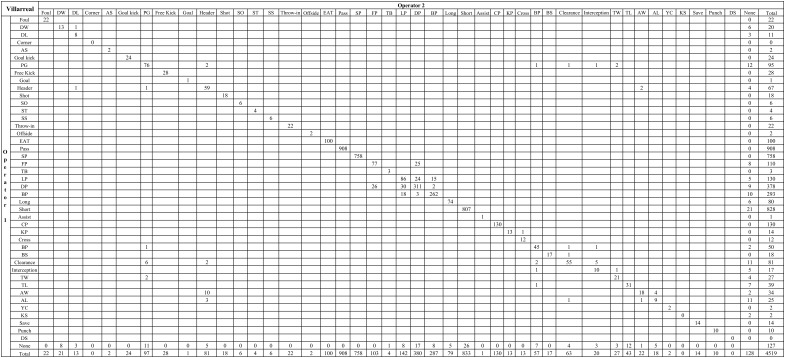
Frequency of Real Madrid’s events recorded by the two independent operators in two collections. DW, dribble won; DL, dribble lost; AS, attacking shift; PG, possession gained; SO, shots off target; ST, shot on target; SS, shot saved; EAT, enter into attacking third; SP, successful pass; FP, forward pass; TB, through ball; LP, lateral pass; DP, diagonal pass; BP, backward pass; CP, consecutive pass; KP, key pass; BP, blocked pass; BS, blocked shot; TW, tackle won; TL, tackle lost; AW, aerial won; AL, aerial lost; YC, yellow card; KS, keeper sweeper; DS, deflected save.

Table 4 showed that there were in total 2,619 events agreed by Operator 1 within two collections for Real Madrid, and 1,948 for Villarreal. The Kappa values for the events of two teams were 0.91 and 0.93. While Table 4 showed that there were in total 2,781 events agreed by Operator 2 within two collections for Real Madrid, and 1,953 for Villarreal. The Kappa values for the events of two teams were 0.91 and 0.87. These results demonstrated a very good intra-operator agreement (see Supplementary Tables 1–4).

**Table 4:** Agreement of team events coded by intra-operators.

		Agreement	First data collection		Second data collection		Kappa value
			Total	Disagreed	Total	Disagreed	
Operator 1	Real Madrid	2,619	2,769	150	2,782	163	0.91
	Villarreal	1,948	2,049	101	2,045	97	0.93
Operator 2	Real Madrid	2,781	2,959	178	2,980	199	0.91
	Villarreal	1,953	2,124	171	2,136	183	0.87

Table 5 shows that the intra-class correlation coefficients (ICC) ranged from 0.98 to 1.00 and the standardized typical errors (TE) varied from 0.01 to 0.15 for different groups of match actions coded by the same operator within two data collections, showing very good intra-operator reliability. The ICC ranged from 0.93 to 1.00, and TE varied from 0.01 to 0.29 for different match actions coded by different operators within two data collections, showing high level of inter-operator reliability. Furthermore, an empirical comparison was made between match statistics provided by OPTA Sports^1^ and Operator 1 and 2 from Champdas Master System, concerning the same match events that both systems comprise (see Table 6). It is shown that generally both operators demonstrated an acceptable agreement with OPTA in all compared variables, expect for a slight discrepancy in short passes.

**Table 5:** Intra-operator and inter-operator reliability of match actions coded within two data collection.

		Real Madrid				Villarreal			
	Variables	Mean (SD)	Change in the mean (CL)	Standardized typical error	ICC	Mean (SD)	Change in the mean (CL)	Standardized typical error	ICC
Operator 1 (1st vs. 2nd)	Attacking	17 (21)	-4.4 (5.4)	0.03	1.00	12 (15)	-0.4 (1.0)	0.09	0.99


	Passing	182 (219)	1.6 (1.5)	0.01	1.00	128 (161)	-0.1 (1.6)	0.01	1.00
	Defending and goalkeeper	7.4 (6.9)	-0.4 (0.8)	0.14	0.99	12 (11)	-0.3 (1.0)	0.07	0.99
	All actions	64 (142)	0.14 (0.49)	0.01	1.00	48 (102)	-0.35 (0.50)	0.01	1.00
Operator 2 (1st vs. 2nd)	Attacking	17 (19)	-0.3 (1.0)	0.07	1.00	13 (16)	0.0 (0.5)	0.05	1.00


	Passing	182 (221)	2.3 (1.9)	0.01	1.00	129 (163)	0.8 (2.1)	0.02	1.00
	Defending and goalkeeper	7.2 (7.5)	0.40 (0.90)	0.15	0.98	10.8 (9.7)	-0.1 (1.0)	0.14	0.99
	All actions	64 (142)	0.70 (0.64)	0.01	1.00	48 (103)	0.21 (0.58)	0.02	1.00
Operator 1vs. Operator 2	Attacking	34 (40)	-0.1 (2.5)	0.08	0.99	25 (31)	0.8 (1.4)	0.06	1.00


	Passing	364 (441)	0.4 (3.0)	0.01	1.00	257 (323)	1.4 (2.9)	0.01	1.00
	Defending and goalkeeper	14 (14)	-0.4 (3.3)	0.29	0.93	22 (20)	-2.4 (3.9)	0.24	0.95
	All actions	128 (285)	-0.05 (1.31)	0.01	1.00	95 (206)	0.05 (1.57)	0.02	1.00

*1st, First data collection; 2nd, second data collection; CL, 95% confidence limits; ICC, intra-class correlation.*

**Table 6:** Match events provided by OPTA Sports and *Champdas Master System*.

	OPTA	Real Madrid				OPTA	Villarreal			
Variables		*Champdas Master System*					*Champdas Master System*			
		Operator 1-1st	Operator 1-2nd	Operator 2-1st	Operator 2-2nd		Operator 1-1st	Operator 1-2nd	Operator 2-1st	Operator 2-2nd
Shots	28	27	27	26	28	10	9	9	8	8
Shots on target	7	7	7	7	7	4	2	2	2	2
Goals	0	0	0	0	0	1	1	1	1	1
Pass success %	89%	84%	84%	84%	84%	84%	83%	84%	84%	83%
Aerial duel success	45%	43%	44%	44%	48%	55%	57%	56%	61%	59%
Dribbles won	8	12	9	10	12	7	11	8	9	12
Tackles	19	19	16	25	23	14	13	13	14	15
Passes	650	620	623	621	626	467	455	453	455	459
Crosses	44	40	40	41	43	7	6	6	6	7
Through balls	0	1	1	1	1	2	1	2	2	2
Short passes	574	495	499	589	592	393	357	356	414	421

*1st and 2nd stand for the first and second match data collections.*

## Discussion

This study has examined the validity and the inter- and intra-operator reliability of *Champdas Master System* operated by different well-trained operators, who were unaware of study purpose. The validation process of this system is important for scientific acknowledgment and credibility. From professional football coaches’ evaluation, match variables included in the system had high levels of content validity. Operators separately coded more than 4,000 events in each data collection, which was higher than the values from previous studies applying other systems such as Prozone, OPTA and Digital.Stadium (Bradley et al., 2007; Liu et al., 2013; Beato et al., 2018). Moreover, results reported in this study showed that *Champdas Master System* had high levels of absolute and relative reliability. This reveals that the system is capable of measuring football match events reliably and provide more technical-tactical performance details than its peer systems.

A practical measure with high validity must have high reliability in the meantime. While a measure with high reliability may have low validity. But only the valid and reliable performance indicators can be reliable used in sports performance profiling (O’Donoghue, 2007; McGarry et al., 2013). Therefore, the study initially examined the validity of performance indicators by evaluating experts’ opinions according to the previous literature (Trniniæ et al., 2000; Hraste et al., 2008; Larkin et al., 2016). Based on the high values of Aiken’s V calculated for twenty professional coaches’ responses to the pertinence (0.84 ± 0.03) and definition (0.85 ± 0.03) of match variables, it was revealed that the variables comprised in the system have appropriate operational definitions and are able to represent match events and player actions during data gathering and analysis.

As previously argued, valid operational definition is not sufficient to guarantee a reliable observation, because it is frequent that human error affects the repeatability of the data. (Williams and O’Donoghue, 2006; O’Donoghue, 2007; Beato et al., 2018). Therefore, the current study verified that after rigorous training and large quantity of practice using *Champdas Master System*, operators could achieve high intra- and inter- operator reliability when coding live football match events, and the data provided were reliable, which was supported by the high Kappa values, high intra-class correlation coefficients and low standardized typical errors. The findings were similar to the previously tested Prozone MatchViewer System (Bradley et al., 2007), OPTA Client System (Liu et al., 2013), and Data.Stadium System (Beato et al., 2018). This suggests that the semi-automatic operation errors in collection with the system were extremely limited. Besides, it should be noted that former research focused on the measurements of typical technical-tactical variables that were mainly notated via shortcut keys (Liu et al., 2013). Nonetheless, compared with its counterparts, the current system is operated via both shortcut keys and mouse clicking on a simulated pitch. On the account, the system not only produces more data related to attacking and passing behaviors, but also effectively provides tactical information, considering pitch zones where match performance took place. This allows more comprehensive technical-tactical match statistics for performance analysis and broadcasting purposes.

However, there were several limitations that should addressed. First of all, more matches may be needed to test the generalization of the system and operator reliability. Additionally, it is admitted that occasions of discrepancy existed in some match actions related to passing directions, short/long pass. This phenomenon happens when comparing OPTA Sport and *Champdas Master System*, as well as assessing intra- and inter operators reliability. This may be explained from two perspectives. Primarily, the inconsistence between OPTA Sport and *Champdas Master System* in short pass might be caused by different definition of the length. This is supported by the similar total number of passes both systems recorded in the current match. Moreover, after a round of retrospection with participating operators, we were informed that the disagreements in determining the lengths and directions of passes mainly originated from plotting errors and entry errors. These types of errors were due to the manual marking discrepancy when operating on the miniaturized on-scree pitch. For example, for the passing direction related variables, the system would automatically recognize a pass as lateral pass even if a player made a forward pass (the angle between pass direction and the parallel of sideline is < 15°) to his teammate who is located at the left front of him. Therefore, if operators could not observe or notate properly similar pass directions on the screen as what are actually taking place on the field, disagreements in passing categories would occur. In fact, this issue was also reported in ProZone MatchViewer system (Bradley et al., 2007; Castellano et al., 2014) and Trakperformance system (Burgess et al., 2006; Edgecomb and Norton, 2006; Carling et al., 2008), which used a similar approach. Furthermore, operator’s observation would be affected while gathering data from live TV coverage, as Tenga and Albin (Tenga, 2010) found that camera angles, image sizes and feature film could be blurry when accurate event location is expected. Consequently, more precision problems appear especially when operators are notating positions, distances and angles related match events.

In a word, the largest technical problem was that operators had difficulty in consistently plotting the X/Y coordinates of the events on the miniaturized pitch. In light of these issues, it is suggested that simplification in passing directions be considered. Instead of including diagonal pass (Figure 1), four types of categories could be established with angle interval of 90° (Goes et al., 2019): forward, left and right sideways, and backward. Meanwhile, automatic player tracking instruments should be further developed and integrated in the data collection process so that operators could avoid subjective determination of directions, lengths and outcomes of passes (Beato and Jamil, 2017). Nonetheless, the current results show that the match statistics could be deemed acceptable so long as operators undergo adequate training and practice in maneuvering the system and identifying specific match events.

The quality of data provided plays a prominent role in performance analysis, sport coaching, media reporting, and scientific research (O’Donoghue, 2009, 2014; O’Donoghue et al., 2017). The study reveals a high level of validity and reliability using *Champdas Master System* to measure live football match statistics. From a theoretical and practical perspective, coaches, sport scientists, and media could benefit from the application of the system to gain reliable technical-tactical performance information. Additionally, future studies could evaluate the generalization of system with a larger sample in similar or distinct leagues, as well as under distinct light conditions.

## Conclusion

The analysis of football expert panel opinions evidenced the validity of tactical and technical match performance variables from *Champdas Master system*. Moreover, high Kappa values, high intra-class correlation coefficients and low standardized typical errors demonstrated a high level of intra- and inter-operator reliability using the *system* to collect sampled match events. Although, slight discrepancy was shown in the identification of players’ passing directions, our results suggest that the *Champdas Master system* can be used validly and reliably to collect live football match statistics by well-trained operators. The system and statistics generated could be trustworthy for coaching, academic research and media report.

## Ethics Statement

We state clearly that written informed consent was obtained from the participants of all phases of the study, and the study was approved by the Ethics Committee of the Technical University of Madrid.

## Author Contributions

M-ÁG, BG, and YC designed the experiments. YC, YG, and QY performed the statistical analysis. BG wrote and revised the manuscript. M-ÁG supervised the design and reviewed the manuscript. All authors have made a substantial and direct contribution to manuscript, and approved the final version of the manuscript.

## Conflict of Interest Statement

The authors declare that the research was conducted in the absence of any commercial or financial relationships that could be construed as a potential conflict of interest.

## Footnotes

1 http://www.whoscored.com
